# Positive association of a women’s continuing medical education conference on career advancement and promotion

**DOI:** 10.1080/10872981.2021.1981127

**Published:** 2021-09-17

**Authors:** Shivani Mukkamala, Priscila Rodrigues Armijo, Laura Flores, Sasha K. Shillcutt

**Affiliations:** a Division of Pediatric Anesthesiology, Department of Anesthesiology, Emory University, Children’s Healthcare of Atlanta, Atlanta, Georgia, USA; b Department of Surgery, University of Nebraska Medical Center, Omaha, Nebraska, USA; c Medical School, University of Nebraska Medical Center, Omaha, Nebraska, USA; d Division of Cardiothoracic Anesthesiology, Department of Anesthesiology, University of Nebraska Medical Center, Omaha, Nebraska, USA

**Keywords:** Women in medicine, gender equity, physician support structures, women physicians, continuing medical education

## Abstract

Women physicians are underrepresented in leadership positions across medical specialties. Understanding factors that improve women’s promotion metrics may lead to career and leadership advancement. This study examined if a woman-centered Continuing Medical Education (CME) conference is associated with differences in productivity metrics toward career advancement. The authors conducted a cross-sectional survey study of women physicians attending a national woman-centered CME conference for professional growth, wellness and networking in September 2019. The survey measured promotion metrics achieved in the year prior to the conference and compared them with previous attendees. Of 425 women attendees of the conference, 389 (91.5%) respondents completed the survey. Respondents were divided into two groups for analysis: first time (FT) attendees, and those that attended the conference previously (PV). In the year preceding the survey, PV attendees were more likely to have published a manuscript as first-author or co-author in a peer-reviewed journal (17.5% vs. 9.7%, p = 0.029), given a talk in their area of practice (48.3% vs. 27.9%, p < 0.001) and to have mentored at least one peer (40.8% vs. 27.5%, p = 0.009) and to have asked for a promotion (15.8% vs. 8.6%, p = 0.033) than FT. As compared to first-time conference attendees, women physicians who previously attended a woman-centered CME conference were more likely to achieve career performance metrics including publications and speaking engagements in the preceding year. This study demonstrated a positive association of Women-centered CME conferences in career advancement metrics for women in medicine and suggests further studies on this and other women-centered CME conferences.

## Introduction

The majority of US medical school matriculants have been women since 2017[1]. However, in healthcare, they constitute 3% of chief medical officers, 6% of department chairs, and 9% of division chiefs [2]. Women are underrepresented in medical society achievement awards and in peer reviewed journals authorships [3]. There is disparity in promotion of women in academic medicine [4] as well as salary discrepancies [5]. Factors include lack of mentorship, election to committees, publishing in journals, and speakerships [6,7]. The gender gap has been hypothesized to be related to bias (unconscious and conscious) [8] and lack of support structures [9].

In 2018, Harvard Business Review published that attending a women’s business conference improved promotion along with financial and intellectual gains amongst attendees [10]. We sought to determine if attending a woman’s medical conference had similar measurable association with metrics of career advancement.

## Methods and materials

This cross-sectional survey was approved by University of Nebraska IRB. Physicians attending The Brave Enough 2019 women’s CME conference were administered an electronic Qualtrics® survey electronically before or during the course, September 12–15, 2019. The survey was developed to measure mentorship, authorship, presentations, committee membership, and promotions. The national CME conference focused on promotion of women physicians and discussed gender bias, leadership, peer support, burnout, and well-being. There were two cohorts: first-time (FT) attendees and those who attended the same conference previously (PV) – 2018 or 2017. Pearson chi-square or Fischer’s chi-square test was used for categorical variables. All statistical analyses were performed using IBM SPSS v26.0 (Chicago, USA).

## Results

Of 425 conference attendees, 389 (91.5%), all women, completed the survey. There were no demographic differences (age, race, specialty, type of practice) between the cohorts (Table 1). Majority of the attendees were between 30–39 (PV 33.1%, FT 42.2%) and 40–49 (PV 60.3%, FT 46.9%), and were Non-Hispanic White (PV 65.8%, FT 60.2%). Respondents were academic (PV 29.7%, FT 20.5%), hospital employed (PV 22.9%, FT 36.9%) or other including private practice (PV 47.4%, FT 42.6%). Top represented medical fields were anesthesiology (PV 12.1%, FT 10.8%), family medicine (PV 9.2%, FT 11.2%), internal medicine (PV 10%, FT 10.8%), and pediatrics (PV 7.5%, FT 16%).

**Table 1.** Demographics of survey respondents

		First Time Attendee (FT)	%	Previous Attendee (PV)	%	
Age	30–39 years	114	42.40%	40	33.30%	P = 0.160
	40–49 years	126	46.80%	72	60.0%	
	50–59 years	26	9.70%	7	5.80%	
	60–69 years	3	1.10%	1	0.80%	
What best describe your racial/ethnic background?	Asian, Pacific Islander	12	4.5%	5	4.2%	P = 0.999
	South Asian or Indian American	38	14.1%	13	10.8%	
	East Asian or Asian American	10	3.7%	5	4.2%	
	Black, Afro-Caribbean, or African American	14	5.2%	5	4.2%	
	Middle Eastern or Arab American	4	1.5%	5	4.2%	
	Non-Hispanic White or Euro-American	162	60.2%	79	65.8%	
	Latino or Hispanic American	18	6.7%	6	5.0%	
	Other	11	4.1%	2	1.7%	
Are you Spanish, Hispanic, or Latino?	Spanish	2	8.3%	0	0.0%	P = 0.727
	Hispanic	12	50.0%	4	57.1%	
	Latino	10	41.7%	3	42.9%	
What is your level of training?	Attending physician	261	97.0%	117	97.5%	p = 0.976
	Non-practicing physician	8	3.0%	3	2.5%	
Where do you PRIMARILY practice?	Academic practice	55	20.5%	35	29.7%	p = 0.058
	Hospital owned practice	99	36.9%	27	22.9%	
	Government practice (VA)	8	3.0%	5	4.2%	
	Other	106	39.6%	51	43.2%	
What is your PRIMARY medical specialty?	Anesthesiology	29	10.8%	18	12.1%	P = 0.086
	Dermatology	7	2.6%	4	2.8%	
	Emergency Medicine	12	4.5%	3	2.5%	
	Family Medicine	30	11.2%	11	9.2%	
	Internal Medicine	29	10.8%	12	10.0%	
	Neurology	6	2.2%	1	0.8%	
	Obstetrics-gynecology	30	11.2%	8	6.7%	
	Ophthalmology	3	1.1%	4	3.3%	
	Pathology	4	1.5%	2	1.7%	
	Pediatrics	43	16.0%	9	7.5%	
	Physical Medicine and Rehabilitation	2	0.7%	2	1.7%	
	Psychiatry	6	2.2%	8	6.7%	
	Radiology	11	4.1%	2	1.7%	
	Surgery	14	5.2%	12	10%	
	Other	42	15.7%	24	20.0%	

The largest differences between PV and FT attendees were in the promotion metrics category (Table 2). In the past year, PV attendees were statistically more likely to have published a manuscript in a peer-reviewed journal (PV 17.5%, FT 9.7%, p = 0.029), to have given a presentation in their area of medical practice (PV 48.3%, FT 27.9%, p < 0.001), to have mentored a peer (PV 40.8%, FT 27.5%, p = 0.009), and to have asked for a promotion (PV 15.8%, FT 8.6%, p = 0.033). In the peer support, and career fulfillment categories, there were no statistical differences between the two groups.

**Table 2.** Reported promotion metrics and mentorship

	No		Yes		P-value
	N	%	N	%	
Promotion Metrics					
Published an abstract locally, regionally, nationally, or internationally					p = 0.102
FT	242	90.0%	27	10.0%	
PV	101	84.2%	19	15.8%	
Published a manuscript as first-author or co-author in a peer-reviewed journal					*p = 0.029*
FT	243	90.3%	26	9.7%	
PV	99	82.5%	21	17.5%	
Given a local, regional, national, or international talk IN MY MAIN area of practice (specialty)					*p < 0.001*
FT	194	72.1%	62	27.9%	
PV	62	51.7%	58	48.3%	
Given a local, regional, national, or international talk OUTSIDE MY MAIN area of practice					p = 0.125
FT	243	90.3%	26	9.7%	
PV	102	85.0%	18	15.0%	
Mentored at least one peer					*P = 0.009*
FT	195	72.5%	74	27.5%	
PV	71	59.2%	49	40.8%	
Received a pay raise					p = 0.204
FT	209	77.7%	60	23.3%	
PV	100	83.3%	20	16.7%	
Asked for a promotion					*p = 0.033*
FT	246	91.4%	23	8.6%	
PV	101	84.2%	19	15.8%	
Received a promotion					p = 0.882
FT	239	88.8%	30	11.2%	
PV	106	88.3%	14	11.7%	
Been nominated to serve on a committee/board					p = 0.217
FT	181	67.3%	88	32.7%	
PV	73	60.8%	47	39.2%	
Served on a committee/board					p = 0.489
FT	138	51.3%	131	48.7%	
PV	57	47.5%	63	52.5%	
Been nominated for an award					p = 0.289
FT	235	87.4%	43	12.6%	
PV	100	83.3%	20	16.7%	

## Discussion

Previous studies demonstrated that women physicians lack mentorship, an important part of leadership, advancement and promotion [6,11]. This study sought to determine if attending women-centered CME conferences is associated with promotion metrics. It revealed that women that previously attended the conference were more likely to report having achieved measurable promotion metrics such as publishing a manuscript, presenting academic work, or asking for a promotion. Additionally, attendees were more likely to mentor peers.

Woitowich et al. recently studied the impact of women centered medical conferences on participants and non-participants [12]. They reported participants were more likely to hold leadership positions and to have received professional accolades in the past year. Non-participants were more likely to be primary caregivers for children or seniors. They solicited respondents through personal email and social media accounts which may have led to selection bias. The study did not review outcomes of a particular conference, rather it looked at attendance of any woman-centered conference. Our survey, while similar, mitigated selection bias by studying the population of attendees of only one conference.

Gender bias in medicine has been suggested in literature to influence the qualifications and opportunities women physicians often lack to be considered for promotion [11,13,14]. Women-specific CME conferences can provide educational programming on institutional bias and may provide opportunities for mentorship. This study provides evidence to suggest that education received along with connections may be associated with women seeking opportunities for promotion and to achieve promotion metrics.

As mentioned by Woitowich et al., barriers to attend women-centered CME conferences include cost and personal responsibilities [12]. Personal and institutional support to attend such conferences could include financial support, protected time to attend, or support from conference organizers to provide family-support structures onsite.

All study participants implicitly had the time, finances, and social support to attend and thus may not represent all women physicians. They may be inherently motivated for promotion, introducing selection bias. The number of academic physicians in each group was not significant nor was the age distribution; however, a trend towards more academic physicians and more 40–49 year olds in PV could be factors for promotion differences. Attendees in academics and further in their careers may have more time and motivation to reach metrics. We did not ask the attendees how many years separated the previous conference attended (2018 or 2017), and if other similar conferences were attended by either group. Another study limitation included causality, as this was not investigated in the survey study.

## Conclusions

This study revealed that women physicians who attended a women-centered CME conference were more likely to achieve promotion metrics compared to first-time women attendees of the conference. Professional conferences focused on career advancement of women physicians may represent a tool to promote women leaders in medicine.
